# Application of Semipermeable Membranes in Glucose Biosensing

**DOI:** 10.3390/membranes6040055

**Published:** 2016-12-14

**Authors:** Tanmay Kulkarni, Gymama Slaughter

**Affiliations:** Bioelectronics Laboratory, Department of Computer Science and Electrical Engineering, University of Maryland Baltimore County, 1000 Hilltop Circle, Baltimore, MD 21250, USA; tank1@umbc.edu

**Keywords:** glucose biosensor, semipermeable membrane, glucose oxidase, pyrolloquinoline quinone glucose dehydrogenase, polypyrrole, poly(3,4-ethylenedioxythiophene, cellulose acetate

## Abstract

Glucose biosensors have received significant attention in recent years due to the escalating mortality rate of diabetes mellitus. Although there is currently no cure for diabetes mellitus, individuals living with diabetes can lead a normal life by maintaining tight control of their blood glucose levels using glucose biosensors (e.g., glucometers). Current research in the field is focused on the optimization and improvement in the performance of glucose biosensors by employing a variety of glucose selective enzymes, mediators and semipermeable membranes to improve the electron transfer between the active center of the enzyme and the electrode substrate. Herein, we summarize the different semipermeable membranes used in the fabrication of the glucose biosensor, that result in improved biosensor sensitivity, selectivity, dynamic range, response time and stability.

## 1. Introduction

Diabetes mellitus is the seventh leading cause of death in the US. Diabetes is broadly classified into two types: type I and type II. Type I diabetes is a result of insufficient insulin production by the pancreas, whereas type II diabetes is due to the body’s inability to use the insulin that is produced, hence the name insulin resistance is used to refer to type II diabetes. Currently, individuals with diabetes are able to monitor their blood glucose levels using a glucometer or a continuous glucose monitor (CGM) in order to prevent further complications such as blindness, ketoacidosis, stroke and even amputation. While the National Institute of Health, the American Diabetes Association and Centers for Disease Control and Prevention are working closely together to find a cure for diabetes, several approaches to “cure” diabetes have been proposed. Some of these approaches include pancreas transplantation, islet cell transplantation, artificial pancreas development and genetic manipulation [1,2,3,4]. These approaches are still in their early stages and possess a lot of challenges. Blood glucose monitoring on a timely basis is the current optimal solution to keep blood glucose levels under control.

**Figure 1:**
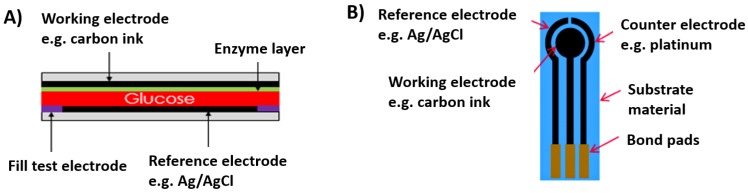
Glucose biosensing principles. (**A**) Coulometric; and (**B**) Amperometric glucose biosensor.

Blood glucose monitors consist of a glucose transducer and electronics that display blood glucose level information in mg/dL. The glucose transducer is an analytical device that converts the chemical energy in glucose to electrical energy and, when coupled with a potentiostat circuit, it is then capable of measuring and displaying the glucose concentration in blood. These traditional glucose monitors consist of a potentiostat circuit which is battery operated, thereby making blood glucose monitors bulky. Various glucose biosensors are available on the market today, which mostly operate based on the principles of coulometric or amperometric electrochemical detection methods [5]. While the coulometric principle relies on the measurement of the total charge necessary to oxidize a finite amount of glucose, the amperometric principle measures the steady state current produced from a finite volume of glucose being oxidized. Typically, a columetric-based biosensor employs a test strip as depicted in Figure 1, consisting of a fill test electrode that fills the test strip with the glucose substrate, which is then oxidized by a glucose-selective enzyme and the amount of charge required to oxidize the glucose substrate is measured when a potential is applied between the working and the reference electrode via the battery operated potentiostat. The measured charge is proportional to the glucose concentration.

On the other hand, the amperometric-based glucose biosensor uses a glucose selective enzyme at the working electrode to oxidize the glucose, which results in the release of electrons. The steady state current is measured by applying a potential between the working electrode and the reference electrode to decompose the hydrogen peroxide produced by the oxidation of glucose. Blood volume as small as 0.3 µL is sufficient for glucose sensing. Since blood glucose monitoring via the use of glucometers requires frequent finger pricking, this can be tedious and painful at times. A completely non-invasive GlucoWatch^®^ G2 Biographer (Cygnus, Redwood City, CA, USA) glucose monitoring device relies on reverse iontophoresis principle for measuring glucose levels. It measures glucose in interstitial fluid. The negative charge of the skin at buffered pH causes it to be permselective to cations such as sodium and potassium ions, allowing iontophoresis that causes electroosmosis, by which neutral molecules, including glucose, are transported across the skin. However, due to the discrepancies in the glucose readings resulting from the interference of sweat, the use of GlucoWatch was discontinued, leaving the glucometer and CGM devices as the most commonly used glucose biosensors for blood glucose monitoring.

**Figure 2:**
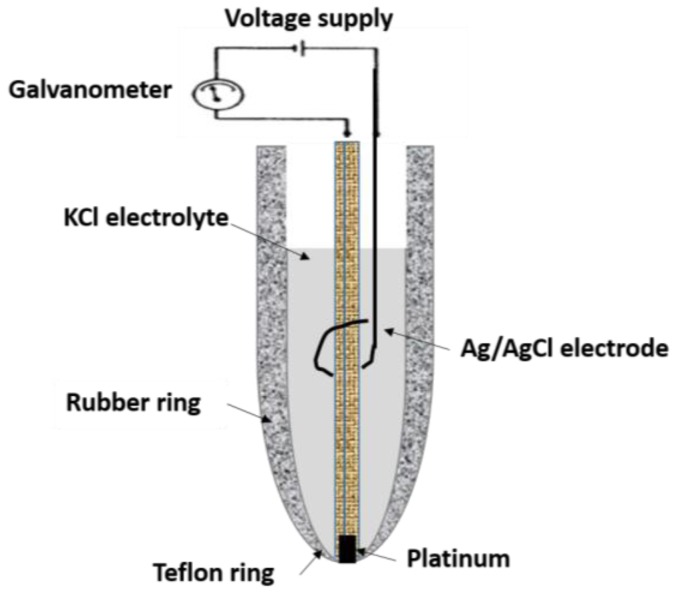
The first oxygen electrode developed by Clark in 1962.

Although there has been a significant progress in the development of glucose sensors that are more compact and easy to use, drawbacks such as calibration issues, bulkiness of the device, warm-up period and the dependence on battery to drive the potentiostat circuit still remain. Therefore, significant research is underway to design novel glucose biosensors that are self-powered [6]. Focus is on improving the sensing parameters using various glucose selective enzymes along with the use of mediators and semipermeable membranes. In addition, the use of semipermeable membrane has gained considerable attention due to its advantages in improving the sensitivity and selectivity of glucose biosensors. Unlike mediators, which improve the electron transfer between the enzyme and the substrate at the cost of complexity and selectivity. Glucose biosensors with semipermeable membranes have been demonstrated to enhance the dynamic range along with sensitivity and selectivity [7]. The semipermeable membrane encapsulates the enzyme as well as provides a microenvironment conducive to maintaining the enzyme’s viability, thereby improving the stability of glucose biosensors. The first glucose biosensor with a semipermeable membrane was developed in 1962 by Leland Clark and Champ Lyons [8]. Figure 2 depicts the schematic illustration of the glucose biosensor composed of an oxygen electrode, an inner oxygen semipermeable membrane, a thin layer of glucose oxidase (GOx) as the glucose selective enzyme, and an outer dialysis membrane. It measures the decrease in oxygen concentration, which is directly proportional to the glucose concentration. Further simplifications in the design of this particular glucose biosensor were made by Updike and Hicks, wherein they immobilized GOx in a polyacrylamide gel on the oxygen electrode in order to create a microenvironment that stabilizes GOx [9,10].

## 2. Generations of Glucose Biosensors

**Figure 3:**
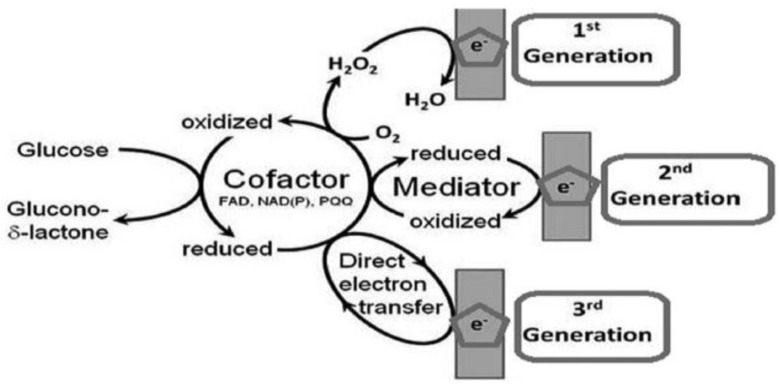
A schematic of the reactions governing different glucose sensor generations [23].

Figure 3 provides a schematic diagram of the various generations of glucose biosensors. First-generation glucose biosensors consist of a natural oxygen substrate and rely upon the detection of hydrogen peroxide production, which is directly proportional to the glucose concentration. The major advantage of the first-generation glucose biosensor is that the measurement process is straightforward [11]. However, a huge drawback of these devices is the performance of the biosensor in the presence of interfering species. Hydrogen peroxide is decomposed at a potential of 700 mV, at which other interfering species such as ascorbic acid and uric acid present in blood are also readily decomposed [12]. This greatly reduces the selectivity of the glucose biosensors and often results in false glucose readings. Moreover, the second-generation glucose biosensors rely heavily on mediators and overcome some of the drawbacks of the first generation glucose biosensors. Mediators are chemical species that carry electrons from the active centers of the enzyme to the electrode. Some of the common mediators employed are ferrocene, thionine and methylene blue. While the mediators are reduced when the glucose oxidation reaction proceeds, these reduced mediators are then re-oxidized at the surface of the electrode thus, providing an amperometric output signal and the re-oxidized mediators can be reused in the reaction [13,14,15,16]. Further, by “electrically wiring” redox polymer to the enzymes, electrons could be shuttled from the enzymes to the transducer [17]. In spite of the use of mediators, these glucose biosensors still suffer from the effects of interfering species such as ascorbic acid and uric acid. Along with the toxicity of some of the mediators, biocatalytic destruction of potential interferences has been observed [18,19,20]. In order to overcome the complications and complexity of employing mediators in biosensor design and development, third-generation glucose biosensors were developed. These biosensors are mediatorless and employ direct electron transfer mechanism between the active center of the enzymes and the electrode surface. In these biosensors, a highly conductive substrate is modified such that there is no need for a mediator. However, only a few enzymes such as pyroloquinoline quinone glucose dehydrogenase (PQQ-GDH) and GOx have been reported to achieve direct electron transfer [21,22,23]. These biosensors have been miniaturized using novel microfabrication technology and thus, found their application in in vivo blood glucose monitoring.

In order to develop an optimal and efficient glucose biosensor, it is important to understand the following eight key characteristics of a glucose biosensor:

1. Accuracy: The ability of the glucose biosensor to produce the output reading close to the true value. The sensor needs to be accurate since any discrepancies can result in drifted glucose readings, which can prove to be fatal.
2. Sensitivity: Sensitivity is determined from the slope of the calibration curve. It is a measure of the change in the biosensor’s output current over the change in glucose concentration. An ideal biosensor will exhibit high and constant sensitivity.
3. Selectivity: Blood is a complex matrix and consists of components other than glucose. Interference species such as ascorbic acid and uric acid and competing species such as fructose, xylose, sucrose, and galactose are present in the body. An ideal glucose biosensor will be highly selective towards glucose compared to the other interfering and competing species.
4. Dynamic range: An ideal glucose biosensor must have a wide dynamic range. Dynamic range is defined as the range of glucose concentration over which the sensor produces linear response. It is essential for a glucose sensor to detect hypoglycemic glucose (<70 mg/dL) levels as well as hyperglycemic glucose (>100 mg/dL) levels along with the normal glucose levels (70–100 mg/dL).
5. Testing volume: From the point of view of patient convenience, an ideal biosensor should be able to operate with a minimal amount of blood sample. Initial glucose biosensor designs required approximately 30 µL of blood. But with advancement in microtechnology and improvement in the design of glucose biosensors, total volume of blood required for testing is presently as low as 0.3 µL.
6. Response time: An ideal biosensor should have fast response time. The response time varies for various glucose biosensors. It ranges between 3 and 60 s. Since the glucose concentration is proportional to the steady state current, it is essential for a sensor to reach steady state response as quickly as possible.
7. Calibration: This is a very important characteristic of a glucose biosensor. It is a measure of the stability of the glucose biosensor. An ideal glucose biosensor should not require frequent recalibration. It should be able to detect glucose for days, sometimes up to months without recalibration. However, current glucose biosensors need calibration whenever new batch of test strips are used.
8. Specificity: This refers to the ability of the glucose biosensor to correctly determine the glucose concentration in the blood sample. The choice of enzyme plays an important role in determining the specificity of the glucose biosensor. At times, the enzyme will be specific to certain functional group instead of an individual analyte. An ideal glucose sensor will have high specificity.

Due to the inconveniences of the present glucose monitoring devices, self-powered glucose biosensors are being developed [6,24]. The success of such systems depend upon glucose biosensors that are of the best quality. One of the factors enhancing glucose biosensor key characteristics are semipermeable membranes employed to encapsulate enzymes as well as to screen against interfering species.

## 3. Semipermeable Membranes

**Figure 4:**
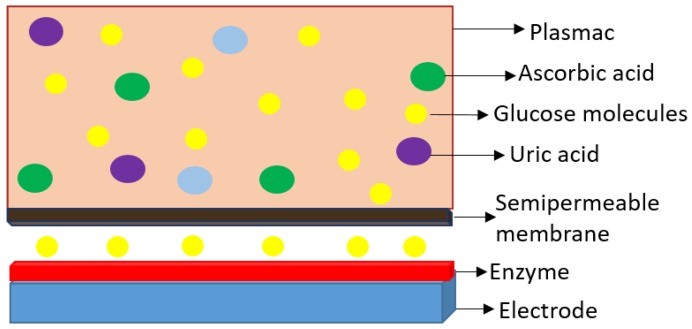
Basic working principle of semipermeable membrane.

The semipermeable membrane used in biosensing applications is a biological or synthetic membrane that allows preferential passage of certain analytes (e.g., molecules and ions) based on size and/or net charge via diffusion. This thereby limits the diffusion of unwanted analytes that could potentially interfere with the desired chemical reactions with the biorecognition element (e.g., enzyme) as shown in Figure 4. Many factors such as the concentration of the analyte; size, net charge, membrane pore size, temperature and pressure govern the passage of the analyte [25]. Permeability of the membrane plays a significant role in prohibiting passage of undesired molecular and/or ionic species. This is governed by the membrane chemical property, thickness and pore size. A glucose biosensor operating in vivo is often subject to a variety of chemical species in plasma that can foul and/or interfere with the chemical reaction occurring on the active site of the bioelectrode, thereby resulting in a decrease in the selectivity of the biosensor [26]. Additionally, biosensors employ semipermeable membranes to prohibit these competing and non-competing analytes from diffusing through to the biorecognition element, thereby, allowing the passage and detection of glucose molecules only. The decomposition of ascorbic acid at 700 mV results in the release of two electrons and corresponding positive ions, which affect the measured current, which often leads to incorrect glucose readings. Thus, semipermeable membranes are necessary to improve the selectivity of these glucose biosensors. Permselective membranes such as cellulose acetate, polyaniline, polypyrrole are based on size exclusion, whereas semipermeable membranes such as nafion, poly(vinylpyridine) and poly(ester-sulfonic acid) are based on charge exclusion [27]. These polymer films are usually solvent-cast or electropolymerized. Table 1 provides chemical structures of the most commonly employed semipermeable membranes used in biosensing applications.

## 4. Non-Enzymatic Glucose Biosensor Membranes

Non-enzymatic glucose biosensors employ precious metals as catalysts to oxidize glucose. Realizing the ability of precious metals such as platinum, gold, copper and palladium to directly electrooxidize glucose, significant research has been conducted in fabricating glucose biosensors using these precious metal catalysts [28,29,30,31]. Bare metal electrodes were initially constructed as glucose sensors. Upon glucose oxidation in the presence of these metal electrodes, it was observed that poisonous metal oxides were formed on the surface of the electrode, which affected the stability and sensitivity of the glucose electrode [32]. Moreover, due to limited reserves of these precious metals, these metals became cost-ineffective for use as bioelectrodes. As a result, bi-metallic catalysts were used in place of mono-metallic catalysts to oxidize glucose [33,34]. Although this overcame the drawbacks of cost-effectiveness and poisonous metal oxide byproducts, sensor parameters such as sensitivity, selectivity and dynamic range remain a challenge. In order to improve the key characteristics of the glucose sensor, semipermeable membranes were employed to coat these electrodes.

Li et al. developed a non-enzymatic glucose biosensor based on glassy carbon electrode modified with hollow nanoparticle (NP) chains of platinum on porous gold nanoparticles in a chitosan membrane [35]. These porous membranes exhibited a highly stable and large surface area for entrapment of platinum hollow nanoparticles. A linear dynamic range from 3 to 7.7 mM with a detection limit of 1 µM was observed. Liu et al. demonstrated a glassy carbon electrode modified with NiO hollow spheres in the presence of chitosan [36]. Fast response time, an essential characteristic of a glucose biosensor, was as achieved by this system (less than 3 s). A low detection limit of 0.3 µM at a signal to noise ratio (SNR) of 3 was calculated. The chitosan membrane was employed as the semipermeable membrane to selectively detect glucose in the presence of other interfering species such as uric acid (UA), ascorbic acid (AA) and dopamine. A huge improvement from previous work was demonstrated in the sensitivity of the non-enzymatic glucose biosensor by Wang et al., wherein another glassy carbon electrode was modified by electrodepositing dendritic copper–cobalt nanostructures (Cu–Co NSs) followed by surface modification by a reduced graphene oxide–chitosan nanocomposite [37]. Such a sensor displayed a dynamic range from 0.015 to 6.95 mM with a sensitivity of 1.921 mA/cm^2^·mM and a detection limit of 10 μM [37]. Shen et al. employed a bi-metallic compound of Pd-Au clusters coated with multiple layers of chitosan [38]. The multiple chitosan layers prevented the Pd-Au cluster from leaching out, thereby improving the stability of glucose sensor. Recently, a novel one step co-deposition of nanocomposites of nickel nanoparticle–attapulgite-reduced graphene oxide (Ni NPs/ATP/RGO) on glassy carbon electrodes was demonstrated by Shen et al. [39]. The sensor was designed to detect lower glucose concentration and exhibited a dynamic range from 1 µM to 710 µM and a detection limit of 0.37 µM [39]. A relatively high sensitivity of 1.4144 mA/mM·cm^2^ and high selectivity amidst interfering species such as UA, AA and 4-Aminophenol was demonstrated. As a result, chitosan membrane-based glucose sensors have gained a tremendous amount of attention due to their advantages in improving the sensitivity and selectivity characteristics of biosensors, as well as being biocompatible and biodegradable.

Apart from chitosan, polymer-based semipermeable membranes are commonly employed in the design of biosensors and significant research has been conducted on exploiting their properties to fabricate a novel optimal glucose biosensor. One such work was conducted by Becerik et al., in which bimetals Pt-Pd layered with a conductive polymer and polypyrrole film [40] were employed. It was observed that polypyrrole film porosity improved the surface area showing higher activity towards glucose oxidation compared to bi-metal electrodes without the conductive polymer film. Jiang et al. reported on the fabrication of a glucose biosensor utilizing electrochemical deposition of Ni(OH)_2_ onto carbon nanotube/polyimide (PI/CNT) membrane [41]. The fabricated sensor exhibited a sensitivity of 2.071 mA/mM·cm^2^ along with a detection limit of 0.36 µM at SNR of 3. The electrodeposition of Ni(OH)_2_ onto carbon nanotube/polyimide (PI/CNT) membrane exhibited long-term stability and good reproducibility when operating in human serum and clearly resulted in an improvement in sensitivity compared to the chitosan membrane employed [41]. Conductive polymers, such as polyaniline (PANI) are also being employed as semipermeable membranes to screen against the interfering species such as uric acid, ascorbic acid and acetaminophen. Ahammad et al. employed gold nanoparticles adsorbed onto glassy carbon electrode modified with PANI [42] as the semipermeable membrane. Using electrochemical impedance spectroscopy technique, they were able to detect glucose concentrations from 0.3 mM to 10 mM with a detection limit of 0.1 mM. And indeed, good selectivity towards interfering species uric acid, ascorbic acid and acetaminophen was demonstrated. Another non-enzymatic glucose sensor was fabricated using a core-shell structure of NiCo_2_O_4_@Polyaniline nanocomposite via a facile hydrothermal treatment followed by polyaniline coating [43]. Polyaniline membrane is highly conductive, thereby, resulting in a higher electrocatalytic activity compared to NiCo_2_O_4_ nanoparticles. The sensor exhibited a linear range up to 4.735 mM with a sensitivity of 4.55 mA/cm^2^·mM and a detection limit of 0.3833 µM. While these non-enzymatic glucose biosensors with semipermeable membranes exhibit good biosensing characteristics, the limitations of these biosensors, especially for bio-implantable applications, has shifted the research focus towards enzymatic glucose biosensors that overcome the current limitations of non-enzymatic glucose biosensors.

## 5. Enzymatic Glucose Biosensor Membranes

Enzyme-based glucose biosensors employ naturally occurring enzymes derived from living organisms as catalysts to oxidize glucose. They are low-cost, easily renewable and a clean source of catalysts thus, overcoming some of the drawbacks of non-enzymatic glucose biosensors. However, enzymes are very fragile and easily affected by external conditions such as temperature, pH, pressure and humidity [44]. Efforts are underway to stabilize these enzymes once immobilized on an electrode substrate to improve their lifetime and stability. To improve the overall performance of an enzymatic glucose biosensor various optimization techniques have been explored [45]. One of the technique is to coat the enzyme with a semipermeable membrane. This membrane prevents the enzyme from leeching out thus, improving the device stability. Also, due to various pore sizes of these membranes, various interfering chemical species are segregated thus, improving the selectivity of the glucose biosensor.

### 5.1. Cellulose Acetate-Based Membranes

**Figure 5:**
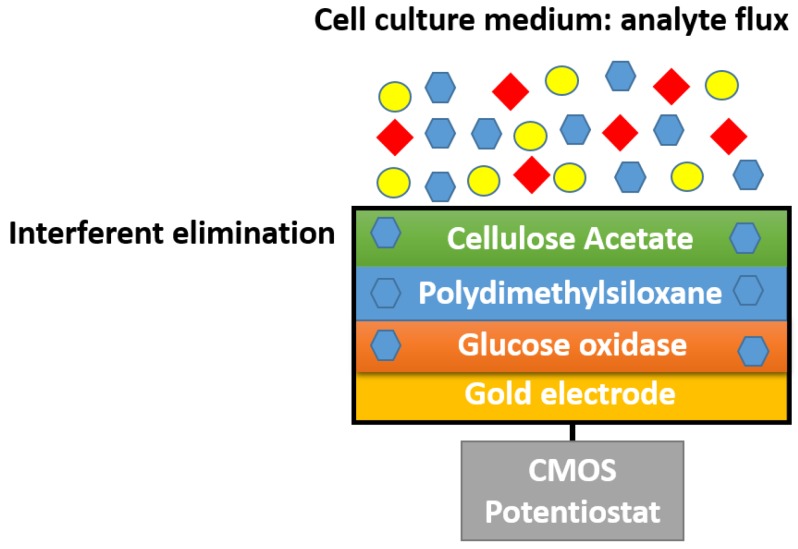
Electrochemical measurement setup for glucose sensing, comprising multi-layer membrane and CMOS potentiostat.

Early research on membrane-based enzymatic glucose biosensors was mostly conducted with collagen membranes. Although collagen films are highly stable and active membranes, they were found to be too thick and fragile [46]. These membranes were extensively explored in the 1970s because of their high perm-selectivity towards anions and the fact that they can be deposited by employing film casting or dip coating method [47]. Significant work has been conducted by Sternberg et al. in which they developed various methods for immobilizing glucose oxidase enzyme on a cellulose acetate membrane [48]. Their work focused on the production of thin and stable membranes. The optimal method involved covalent coupling of bovine serum albumin (BSA) to cellulose acetate membrane and subsequently with the GOx which was then activated with p-benzoquinone. Such work yielded thin membranes of 5–20 µm with high surface activities of 1–3 U/cm³ and stability of up to 3 months. Recently, the cellulose acetate membrane has resurfaced as a semipermeable membrane for enzymatic glucose biosensors. Glucose biosensors consisting of biological and electronic water-based ink containing GOx and conducting polymer blend poly(3,4-ethylenedioxythiophene/polystyrene sulphonic acid (PEDOT/PSS) was thermally inkjet printed on an indium tin oxide (ITO) glass substrate by Setti et al. [49]. This device was encapsulated in a cellulose acetate semipermeable membrane via dip-coating. The glucose biosensor produced a linear response up to 60 mM with a sensitivity of 0.00643 mA/mM·cm^2^. Figure 5 illustrates the two-layer cellulose acetate membrane employed in the development of an electrochemical measurement set up comprising of a glucose biosensor and a complementary metal oxide semiconductor (CMOS) potentiostat [50]. Although the sensitivity of the biosensor was relatively low, the membrane was capable of eliminating ascorbic acid, l-glutathione and l-cysteine, impeding diffusion through the membrane to the biorecognition element, the GOx-modified bioelectrode as shown in Figure 5.

### 5.2. Nafion-Based Membranes

**Figure 6:**
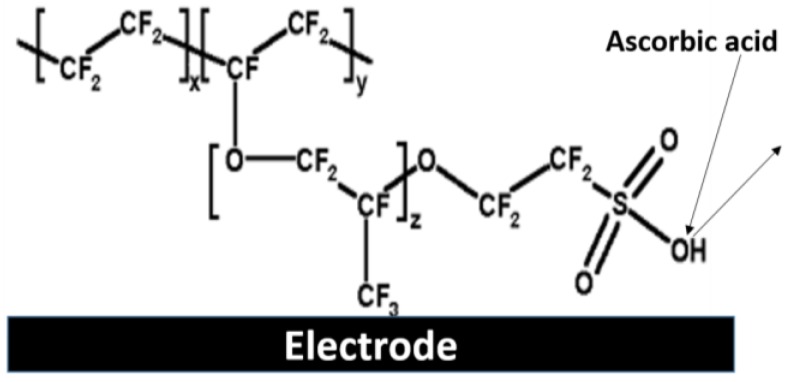
Schematic representation of nafion membrane restricting passage of interfering analyte, ascorbic acid.

Perfluorosulphonic acid polymer, commonly known as nafion is one of the most commonly employed semipermeable membrane in the design and development of glucose biosensors. This perfluorinated cation-exchange polymer with a hydrophobic perfluoro backbone and pendant sulfonic acid groups allows for the permeation of hydrogen peroxide while restricting the passage of anions (e.g., ascorbic acid and uric acid) across the membrane [51]. This thereby reduces electrode fouling and interference by ascorbic acid (as shown in Figure 6) as a result of the negatively charged pendant sulfonic acid groups that prohibit the passage of these negatively charged analytes.

Harrison et al. modified a Pt electrode with GOx followed by nafion coating [52]. The thickness of the nafion membrane was 1.7 µm thick to enable continuous in vitro measurement of glucose in blood at 37 °C. A linear response of up to 28 mM with a response time ranging from 5 to 17 s was observed which was significantly higher than bioelectrodes coated with cellulose acetate membranes. A new mixed membrane material consisting of nafion and laponite gel was used to encapsulate GOx in order to modulate the enzyme loading in the biomembrane. The sensitivity of glucose sensing was found to be directly proportional to the enzyme content in the gel membrane. A GOx to laponite ratio of 3.3 achieved a sensitivity of 132 mA/mM·cm^2^ over a linear dynamic range from 0.01 mM to 20 mM. The effect of interfering species such as ascorbate, urate and acetaminophen was reduced by a factor of 4 with the use of nafion membrane and polyphenol oxidase [53].

Carbon nanotubes have been explored as electrode substrates for their advantages over other metallic and glassy carbon electrodes. Some of the advantages include strong bonding between the atoms and the tubes and extreme aspect ratios thus, improving the conductivity and surface area for enzyme immobilization. Current research has demonstrated that GOx and palladium nanoparticles can be readily co-deposited on a nafion-solubilized carbon nanotube [54]. The fabricated glucose biosensor showed a linear response of up to 12 mM with a detection limit of 0.15 mM with a SNR of 3. The nafion coating eliminated the effects of common interfering species such as uric acid and ascorbic acid. The use of carbon nanotubes as the electrode substrates for enzyme immobilization was very effective and resulted in the exploration of carbon nanowires and a more compact aggregated chain of multi-walled carbon nanotubes (i.e., Buckypaper) as the substrates for high-density enzyme loading.

A self-powered glucose biosensing microsystem was recently fabricated by Slaughter et al., which was powered by a biofuel cell consisting of pyroloquinoline quinone glucose dehydrogenase-modified bioanode and laccase-modified biocathode [6]. A transducer element capacitor connected at the output of charge pump was used to sense glucose via monitoring the charge cycle across it. The bioelectrodes were coated with nafion membrane which prevented the enzymes from leeching out and effectively screening against competing analytes. Such novel system exhibited a stable operation over 97 days in vitro. This novel biosensing system showed promise in effectively screening against interfering analytes because it did not produce the necessary 700 mV required to break down interfering analytes [55]. The presence of interfering analytes has no impact in the glucose readings and thus, results in an improvement in selectivity.

In addition, a glucose biosensor was developed to measure the cerebral glucose levels in order to understand the mechanisms involving insulin and anti-hypertensive drugs regulated in hyperglycemic diabetic rats [56]. This custom-built glucose micro-biosensor was implanted in the striatum. The biosensor comprised of GOx trapped inside a poly 4-vinylpyridine (P4VP) membrane and deposited on Pt electrode. Furthermore, the biosensor was coated with a thin nafion layer. It was observed that the administration of insulin had no significant effect on both hyperglycemic and diabetic rats but the anti-hypertensive drug lowered the glucose levels in the brain. This was the first implementation of a glucose biosensor developed to measure the cerebral glucose levels and thus, shows promise to better understand the complex mechanism of the brain.

Besides showing great promise in screening against negatively charged analytes, nafion has been shown to be ineffective with neutrally charged analytes, such as acetaminophen. To prohibit acetaminophen passage, composite cellulose acetate and nafion membranes have been designed to eliminate the of passage acetaminophen at the cost of poor sensitivity [57]. However, such composite membranes can be useful in different glucose biosensors, except for the first-generation glucose biosensors.

### 5.3. Other Polymer-Based Membranes

**Figure 7:**
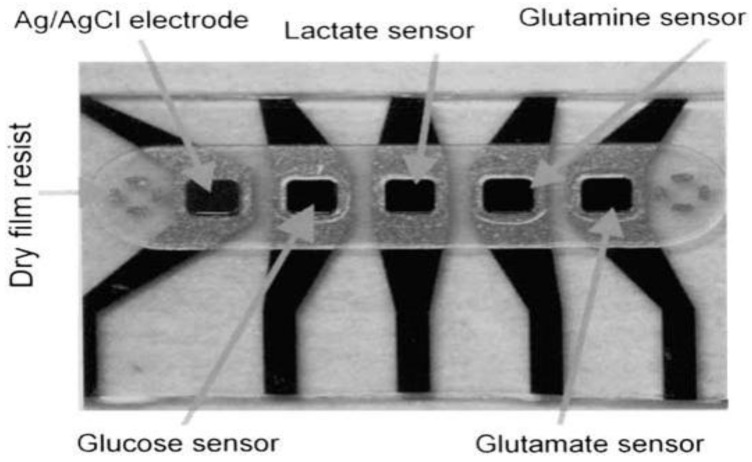
The biosensor array with glucose, lactate, glutamine, and glutamate sensor [60].

Due to the redox-switchable nature of polypyrrole, it was exploited in the design and development of glucose biosensor [58], wherein a glucose biosensor was designed by Ramanavičius et al. In this study, glucose oxidase nanoparticles were encapsulated within a polypyrrole membrane [58]. The incorporation of polypyrrole, a highly conductive polymer, was shown to increase the Michalis-Menten constant (K_M_) and the rate of reaction (K_cat_). An alternative approach was employed to electropolymerized m-phenylene diamine film with GOx, lactate oxidase and glutamate oxidase on a carbon fiber electrode covered with electrometalized ruthenium layer [59]. This biosensor exhibited a relatively small dynamic range of up to 4 mM for glucose with a detection limit of 0.5 µM with SNR of 3. It was operationally stable for over 10 h in a dynamic environment at 36 °C and pH of 7.4. Such a system allowed for characterization of the glucose in vivo, however, it was incapable of detecting normal glucose levels as well as hyperglycemia due to its very narrow dynamic range. Additionally, composite polymer layers such as polyurethane and nafion continue to gain attention in complete screening against interfering species such as ascorbic acid, uric acid, l-cysteine, acetaminophen, dopamine, aspartic acid, glutamine and homovanillic acid [59]. A co-polymer hydrogel consisting of 1,3-diaminobenzene has been used in the design of a novel biosensor array capable of simultaneously sensing glucose, lactate, glutamine and glutamate is shown in Figure 7. The system comprised of a glass chip with integrated biosensor array and a gold electrode to provide electrical continuity. This biosensor system operated over a wide dynamic range of 0.1 mM–35 mM. However, the sensitivity of the biosensor was very low (5–20 nA/mM cm^2^). The biosensor system exhibited an operational stability of a little over 4 weeks and a storage stability of 2 years with less than 0.5 mM in response to interference.

**Figure 8:**
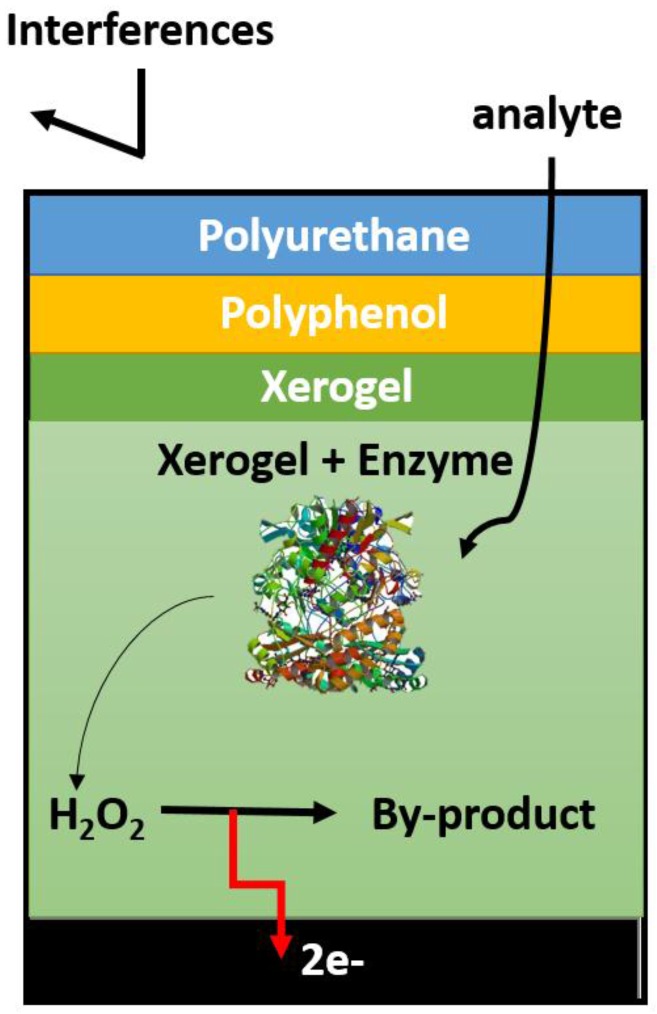
Schematic diagram of a xerogel-based, first-generation amperometric glucose biosensor featuring an enzyme doped and diffusion-limiting xerogel layers and capped with semipermeable electropolymerized polyphenol and polyurethane outer membranes.

A glucose biosensor, in which glucose oxidase enzyme immobilized on Pt electrode and subsequently coated with a permselective membrane poly(4-vinylpyridine-co-styrene) [61] has been demonstrated with dynamic range in the lower glucose concentration range (0.01 mM–1.5 mM) that exhibited a high sensitivity of 30 mA/mM·cm^2^. The membrane employed was found to be successful in the elimination of the effects of ascorbic acid, urate and p-acetaminophen. In order to increase the sensitivity of the glucose biosensor, GOx was immobilized in the presence of BSA on a nano-yarn carbon nanotube followed by coating with epoxy-polyurethane. A 7.5-fold increase in the glucose sensitivity was observed compared to the use of Pt–Ir coil-based electrode and exhibited an operating stability over 70 days. Polyphenol-polyurethane electropolymerization techniques as shown in Figure 8 have been employed with xerogel to encapsulate GOx [62]. The system achieved a linear dynamic range over 28 mM with a very fast response time. The membrane achieved selective glucose sensing in the presence of acetaminophen, ascorbic acid, sodium nitrate, oxalic acid and uric acid. Clearly, the incorporation of various polymer-based membranes have been shown to be critical in improving the biosensor’s selectivity, sensitivity and linear dynamic range.

### 5.4. Chitosan-Based Membrane

Chitin, a naturally occurring chemical, is abundantly available in crustaceans and is known to consist of 2-acetamido-2-deoxy-β-d-glucose. Its immunogenicity is exceptionally low, along with it being a highly insoluble material. Chitosan is the *N*-deacetylated derivative of chitin and is highly biocompatible. As a result, it has become a prominent semipermeable membrane in enzymatic glucose sensors. One of the prominent works that improved the electron transfer rate was demonstrated by Liu et al., wherein GOx was trapped in a composite mixture of carbon nanotube and chitosan resulting in a combination that enabled the enhancement in the direct electron transfer rate (7.73/s) which was more than one-fold increase over GOx-adsorbed on carbon nanotubes (3.10/s) [63]. Moreover, the sensitivity of such biosensor was calculated to be 0.577 mA/mM·cm^2^. The use of chitosan membrane ensured that enzymes stay entrapped, thus improving the stability of the biosensor. Since metal surfaces also possess high affinity towards enzyme immobilization, Zeng et al. immobilized GOx on palladium nanoparticles modified with chitosan membrane [64]. Improvement in biocompatibility and hydrophilicity was observed with a low reaction rate constant ensuring enhanced enzyme affinity to glucose. The sensor exhibited a linear dynamic range from 1 µM to 1 mM with a detection limit of 0.2 µM at SNR of 3 and a sensitivity of 0.031 mA/mM·cm^2^. Ang et al. recently developed a glucose biosensor and characterized it in a fruit [65]. The glucose biosensor was constructed from a Pt electrode modified with glucose oxidase immobilized in a chitosan membrane. There was an observed improvement in the rate of reaction when compared to the system developed by Zeng et al. The limit of detection for this biosensor was observed to be 0.05 mM at SNR of 3. The sensor showed good stability with high enzyme retention activity. It also showed good repeatability and reproducibility with a relative standard deviation of 2.30% and 3.70% in the collected data, respectively. Another application-based glucose biosensor was recently developed, in which Prussian blue modified graphene strings were immobilized with GOx within a biocompatible chitosan layer. A linear dynamic range from 0.01 mM to 1 mM with a response time of less than 3 s was reported [66]. Sensitivity of glucose biosensor was observed to be 0.641 µA/mM·cm^2^. However, this combination resulted in a decrease in the chitosan membrane to completely eliminate or screen against ascorbic acid, uric acid, galactose and acetaminophen.

### 5.5. Poly(2-hydroxyethyl Methacrylate) (pHEMA)-Based Membranes

**Figure 9:**
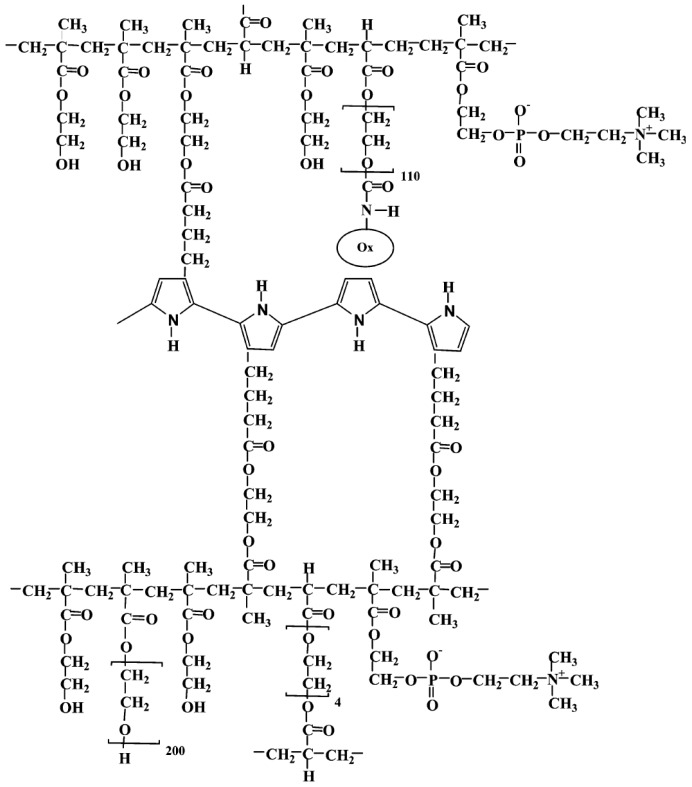
pHEMA and PPy hydrogel membrane comprising of entrapped oxidase enzyme [74]. pHEMA: poly(2-hydroxyethyl methacrylate); PPy: polypyrrole.

Flexible and hydrophilic hydrogels have also been investigated extensively as semipermeable membranes in enzymatic glucose biosensors [67,68]. Hydrogel membranes are made up of mostly water and have been found to be biocompatible. They are commonly used to entrap enzymes in the development of glucose biosensors. A glucose-permeable hydrogel made from crosslinking 8-armed amine terminated poly(ethylene glycol) (PEG) in aqueous solution at room temperature was assayed for biocompatibility in a rat model [69]. Although it was observed that the presence of the cross-linked PEG hydrogel deteriorated and resulted in 34% drop in the biosensor sensitivity when characterized in glucose concentration from 0 to 30 mM, these gels were found to be good candidates for bio-implantable biosensors. Arica et al. characterized the effects of various parameters such as temperature, concentration of hydrogel components and storage life of poly(2-hydroxyethyl methacrylate) (pHEMA)-based hydrogels as semipermeable membranes [70]. GOx was entrapped in the pHEMA membrane through matrix entrapment. It was observed that the affinity of GOx towards glucose decreased substantially. Although membranes with the highest enzyme activity were found to be most permeable, thereby increasing the enzyme content of the membrane adversely affected the biosensor’s activity. The membrane permeability was however, observed to increase at low pHEMA concentrations. Noting the importance of pHEMA gels, glucose biosensors were fabricated using pHEMA, poly(ethylene glycol) and tetra-acrylate and ethylene dimethacrylate [71,72,73,74,75]. Quinn et al. electrically wired GOx to a gold current collector via a redox polymer, which resulted in a 45% ± 28% decrease in the biosensor response at physiological condition [71]. Brahim et al. developed an ‘intelligent’ hydrogel by incorporating polypyrrole (PPy) within the highly pHEMA-based hydrogel to yield a PPy-pHEMA hydrogel [75]. It was observed that the ‘intelligent’ hydrogel retains its hydration and as well as the electroactivity of the conducting polymer, polypyrrole. This PPy-pHEMA hydrogel composite in Figure 9 was employed as a semipermeable membrane in the construction of a dual glucose and lactate biosensors by Guiseppi-Elie et al. [74].

The PPy component provided interference screening capabilities, whereas the pHEMA provided excellent in vivo biocompatibility. A linear dynamic range of 0.10–13.0 mM for glucose and up to 90 mM for lactate was observed. A PEDOT component was used in place of the polypyrrole component to create p(HEMA)-PEDOT membrane that enhanced the stability of the biosensor [73]. The biosensor exhibited stability over 90 days and selectively screened against the competing analyte fructose.

A needle-shaped platinum enzymatic glucose biosensor based on GOx immobilized in a pHEMA membrane that was coated with an outer membrane composed of a pHEMA/polyurethane composite mixture was developed by Shaw et al. [76]. It exhibited long-term stability when operating in 5 mM glucose solution in vitro and suffered no significant loss over a 60 h of continuous operation. Moreover, a linear dynamic range of up to 20 mM glucose was observed.

To overcome some of the drawbacks of GOx, glucose dehydrogenase enzymes are being employed in the development of glucose biosensors. A glucose biosensor was developed by immobilizing glucose dehydrogenase (GDH) and nicotinamide adenine dinucleotide phosphate (NADP^+^) coenzyme on a biocomposite made of graphite powder and polymethacrylate [77]. The procedure was highly reproducible and exhibited stability over 120 days. Table 2 summarizes the glucose biosensor employing various semipermeable membranes that have been employed in the design and development of glucose biosensor membranes.

## 6. Conclusions

Semipermeable membranes play a significant role in improving glucose sensor characteristics. Cellulose acetate membranes were one of the earliest and most commonly used commercial form of reverse osmosis membranes. These membranes have an added benefit of low cost and high tolerance towards chlorine, which is essential since they are more susceptible to biodegradation. They quickly gained significant attention as semipermeable membranes for glucose biosensors. They were typically manufactured as thick membranes, which limit analyte diffusion and were not ideal for glucose biosensing. For use as thin membranes with lower permeability, they are subjected to high pressures, which result in an increase in manufacturing cost. Moreover, they are vulnerable to hydrolysis in the presence of acids and alkalies, which render them inferior to salt rejection. Physiological fluids have fair amount of salt content and that can affect the performance of these membranes when employed in glucose biosensing. With a narrow pH range of 4–8 and a temperature range of 0–35 °C, these membranes fail to operate at physiological temperatures and hence are now being replaced by other semipermeable membranes that have wide operating pH and temperature ranges.

Nafion has been explored by many researchers to entrap enzymes and create a microenvironment to improve the long-term stability of biosensors [78,79]. These membranes overcome the challenges exhibited by cellulose acetate membranes and can operate in wide ranges of pH and temperature (<100 °C) [80]. Although this membrane exhibits great advantages, the biggest drawback is its poor performance under low humidification conditions [81], high oxygen permeability (9.3 × 10^−12^ mol/cm^2^·s) and its susceptibility to membrane fouling, which in turn limits the operational stability of the biosensors. These limitations make nafion membrane an unpopular choice for commercial biosensors [82]. Due to these observed limitations, the research direction shifted towards exploration of other polymer membranes. The highly conductive (2–100 S/cm) and switchable nature of the polypyrrole membrane along with its high thermal stability result in a higher ionic transfer rate [82] than with nafion membranes. This has resulted in its incorporation into various semipermeable membranes. Polyphenols structures, on the other hand, consist of large π-electron configurations that enable them to have great affinity towards enzymes [83], whereas polyurethane films have been used in combination with other polymer membranes to provide flexible but hard membrane structures to shield the bioelectrode from physical damage. Although different polymeric membranes have been used to minimize the effect of interferents, there is still a need for variety of biocompatible materials that can easily screen against interferents and improve biosensor characteristics if they are to be used in vivo. Overall, nafion and chitosan-based membranes have exhibited high biocompatibility in addition to enhancing the sensitivity and selectivity of biosensors. The added benefit of incorporating chitosan-based films is that they exhibit excellent oxygen barrier ability [84] and are biodegradable, non-toxic, inert and hydrophilic [85].

Non-toxicity along with excellent chemical stability has made PPy-pHEMA membranes a common choice to minimize the effects of interferents in glucose biosensing systems. pHEMA semipermeable membranes like chitosan are biocompatible. Moreover, they possess stronger mechanical properties compared to almost all other polymers [86]. Their mechanical properties can be easily improved with bulk polymerization and copolymerization [87,88]. Although these semipermeable membranes exhibit excellent enzyme retaining ability along with complimentary physical properties that improve the performance of biosensors, very rarely have they been implemented in practice due to the increase in complexity of bioelectrode design. Due to the fragile nature of enzymes, the in situ synthesis of these semipermeable membranes is likely to negatively impact the enzymes, thereby altering their properties and possibly denaturing/deactivating them. In most glucose biosensors, the bioelectrodes are first modified with the biorecognition element within a semipermeable membrane followed by an outer membrane coating that is employed to protect the inner membrane and thus, the biorecognition element. The addition of a second or even a multiple semipermeable membrane layers further limits the passage of the desired analyte and thus, affects the overall performance of the glucose biosensors. Although Tipnis et al. demonstrated layer by layer development of multi-layer membrane for glucose biosensing [89], further work needs to be done in optimizing the protocol before it can be use in practice.

The various membranes reviewed have been shown to improve the electron transfer rate along with selectively oxidizing glucose in the presence of common interfering species such as ascorbic acid, uric acid and acetaminophen, fructose. Furthermore, the use of biocompatible semipermeable membranes has been shown to increase successful implantation along with good operational stability in vivo. Although there has been a tremendous improvement in sensing characteristics, long-term stability and operational lifetime remain a challenge to surpass the performance of the already available commercial glucose biosensors. Significant work is underway in which microsystem technology offers a promising future in the glucose biosensor industry, potentially replacing present glucose monitoring systems which are bulky, battery-powered and require frequent recalibration.

## Figures and Tables

**Table 1:** The chemical structures of commonly used semipermeable membranes in biosensing.

**Cellulose Acetate**
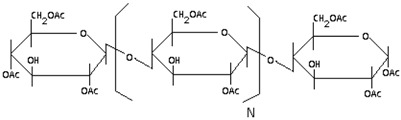
**Nafion**
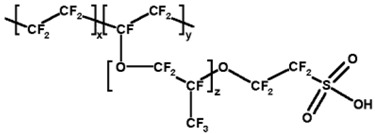
**Polypyrrole**
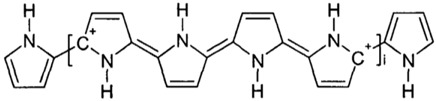
**Polyurethane**
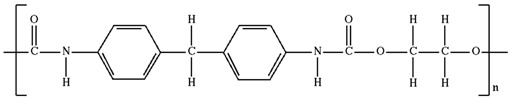
**Chitosan**
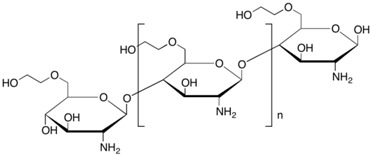
**Poly(2-hydroxyethyl methacrylate)**
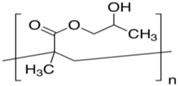

**Table 2:** Summary of Semipermeable Membrane in Glucose Biosensors.

Substrate	Enzyme	Analyte	Membrane	Sensitivity (µA/mM·cm^2^)	Linear Dynamic Range (mM)	Author	Reference Number
Biological- and water-based inks	Glucose oxidase	Glucose	Cellulose acetate	6.43 (µA/M·cm^2^)	Upto 60 mM	Setti, L., et al. (2005)	[49]
Ceramic	Glucose oxidase	Glucose	Polydimethylsiloxane, Cellulose acetate	0.1922 (µA/mM·cm^2^)	Upto 200 mM	Mross, Stefan, et al. (2015)	[50]
Platinum	Glucose oxidase	Glucose	Nafion	176.18 (µA/mM·cm^2^)	Upto 28 mM	Harrison, D., et al. (1988)	[52]
Platinum	Glucose oxidase	Glucose	Nafion	132 (mA/mM·cm^2^)	0.01–20 mM	Poyard, S., et al. (1998)	[53]
Carbon nanotube	Glucose oxidase	Glucose	Nafion	–	Upto 12 mM	Lim, San Hua, et al. (2005)	[54]
Carbon fiber + Ruthenium	Glucose oxidase + Lactate oxidase + Glutamate oxidase	Glucose, Glutamate and Lactate	m-phenylene diamine	–	Glucose (upto 4 mM), Glutamate (upto 0.25 mM), Lactate (upto 1.75 mM)	Schuvailo, O.M., et al. (2006)	[59]
Platinum	Glucose oxidase + Lactate oxidase + Glutamate oxidase	Glucose, Glutamine, Glutamate and Lactate	1,3-Diaminobenzene	Glucose (5–20 (nA/mM·mm^2^)), Lactate (10–40 (nA/mM·mm^2^)), Glutamine (30 (nA/mM·mm^2^)), Glutamate (20–400 (nA/mM·mm^2^))	Glucose (0.1–35 mM), Lactate (0.05–15 mM), Glutamine (0.05–10 mM), Glutamate (0.001–5 mM)	Moser, I., et al. (2002)	[60]
Platinum	Glucose oxidase	Glucose	Poly(4-vinylpyridine-co-styrene)	30 (mA/mM·cm^2^)	0.01–1.5 mM	Poyard, S., et al. (1999)	[61]
Platinum	Glucose oxidase	Glucose	Polyphenol + Polyurethane	354.23 (µA/mM·cm^2^)	≥24–28 mM	Poulos, N.G., et al. (2015)	[62]
Carbon nanotube	Glucose oxidase	Glucose	Chitosan	184.4 (µA/mM·cm^2^)	0–7.8 mM	Liu, Ying, et al. (2005)	[63]
Palladium nanoparticles + graphene	Glucose oxidase	Glucose	Chitosan	31.2 (µA/mM·cm^2^)	0.001–1 mM	Zeng, Qiong, et al. (2011)	[64]
Platinum	Glucose oxidase	Glucose	Chitosan	10.18 (mA/mM·cm^2^)	0.01–15 mM	Ang, L.F., et al. (2015)	[59]
Prussian blue graphite strings	Glucose oxidase	Glucose	Chitosan	641.3 (µA/mM·cm^2^)	0.03–1 mM	Lee, Seung Ho, et al. (2016)	[60]
Gold wire	Glucose oxidase	Glucose	Poly(ethylene glycol) (PEG)	616.11 (µA/mM·cm^2^)	0–30 mM	Quinn, C.A., et al. (1997)	[63]
